# Safe seroconversion after receiving measles, mumps, and rubella live vaccine during guselkumab treatment for psoriasis

**DOI:** 10.1016/j.jdcr.2026.05.006

**Published:** 2026-05-27

**Authors:** Kevin Kim, Carly Kirshen

**Affiliations:** From the Division of Dermatology, Department of Medicine, University of Ottawa, Ottawa, Canada

**Keywords:** guselkumab, IL-23, live-attenuated vaccines, measles, mumps, psoriasis vulgaris, rubella

## Introduction

Biologic therapies target cytokines involved in pathways critical for host defense, raising concern for risk of severe infections. Prospective studies of biologics monitored the frequency and severity of infections to demonstrate safety, and postmarketing surveillance further supported the safety of new biologic therapies. However, these studies avoided the administration of live-attenuated vaccines to mitigate any theoretical risk of severe infections from the attenuated pathogens. In consequence, live-vaccine administration is still avoided in clinical practice, and the risk of vaccine-strain infections remain a theoretical risk. Guidelines and expert consensus from professional societies acknowledge the lack of evidence to ascertain this theoretical risk and propose discontinuing the biologics prior to administering the live-attenuated vaccine, then restarting the biologics afterwards.^1^^,^^2^

Herein, we present a case of a 42-year-old man treated with guselkumab for his long-standing plaque psoriasis who received the live-attenuated measles, mumps, rubella (MMR) vaccine and achieved seroconversion with no complications.

## Case report

A 42-year-old man with no past medical history was diagnosed with plaque psoriasis in 2011 and was first seen in the dermatology clinic in 2014, requiring systemic treatment. He eventually required biologic systemic therapy and began guselkumab, an interleukin 23 (IL-23) inhibitor, in April 2020. He achieved complete clearance of their psoriasis without recurrence. On May 6, 2025, their general practitioner administered the live-attenuated MMR vaccine after his IgG titers were nonreactive. The patient experienced no signs and symptoms of vaccine-strain infections; his psoriasis also did not reoccur. The patient’s MMR IgG titers were all reactive on investigations completed in November 2025, confirming successful seroconversion. His psoriasis remains in remission with guselkumab 100 mg, administered every 8 weeks.

## Discussion

The role of individual cytokines in host defense raise concern for immunosuppression and severe infection when inhibited by biologic therapy. For instance, IL-23 differentiates and stimulates Th17 lymphocytes that are critical for host defense against extracellular microbes and fungi, but also promote mucosal defense and tissue integrity.^3^ As such, guidelines and more recent expert consensus recommend interrupting treatment to administer live vaccines due to the increased risk of infection from the live vaccine.^1^^,^^2^ However, available evidence on the incidence and severity of infections induced by live vaccines may not support discontinuing biologics. In their systematic review published in 2017, Croce et al^4^ examined 64 studies reporting on the safety of live-attenuated vaccine administration (ie, MMR, yellow fever, varicella, herpes zoster, typhoid, polio, rotavirus, Bacillus Calmette-Guérin, and smallpox) in patients receiving immunosuppressive therapy for autoimmune diseases, solid organ transplant recipients, and those who underwent bone marrow transplantation in the preceding 2 years. From a total of 21,082 patients, authors identified 32 patients who developed a vaccine-strain infection; most reported a mild course of infection although there were 2 fatalities. One fatality occurred in a 49-year-old woman with rheumatoid arthritis and systemic lupus erythematosus who started methotrexate and dexamethasone 4 days after receiving the yellow fever vaccine.^5^ This case is confounded by a manufacturing error; the administered vaccine came from a specific lot found to be associated with a >20 times risk of yellow fever vaccine-strain infection. The other fatality occurred in a 3-month-old infant who received the Bacillus Calmette-Guérin vaccine after being born to a mother with Crohn Disease who received infliximab treatment throughout the pregnancy.^6^ In addition to rare reporting of vaccine-strain infections and even rarer fatalities, results from a more recent randomized controlled trial published in 2021 suggest live-attenuated vaccines may be safely administered without discontinuing biologics. Curtis et al^7^ randomized 617 patients receiving tumor necrosis factor (TNF) inhibitor therapy to receive the varicella vaccine or placebo and found no incidence of vaccine-strain infection after 1-year follow-up in patients who received the vaccine. This finding is striking for 2 reasons. First, TNF inhibitor therapy is considered more immunosuppressive than other biologics for the treatment of psoriasis. Second, 58.2% of all patients, and 59.1% of vaccine recipients, were also receiving adjuvant methotrexate or glucocorticoids; this suggests patients were further immunosuppressed than expected from TNF inhibitor monotherapy. There are no reported cases of live-attenuated vaccination in patients on current IL-23 inhibitor therapy.

Current guidance acknowledges the scarce evidence of vaccine-strain infection but still recommends discontinuing biologics for 2 to 3 half-lives, or 4 weeks, before and after the administration of live vaccines.^1^ This may be inconsequential for many patients, but there is an inevitable incurred risk with this approach. Discontinuing biologics increases the risk of disease relapse, restarting treatment may result in decreased efficacy or increased time to achieve the previous effect.^8^

In summary, we present a 42-year-old man with long-standing psoriasis, in remission with guselkumab, who received the live-attenuated MMR vaccine and achieved seroconversion without any complications. Available evidence of harm and recent evidence of safety suggest live-attenuated vaccines may be safely administered in most patients receiving biologic therapy for psoriasis. This case supports the biological plausibility that IL-23 inhibition may not significantly impair MMR vaccine safety or immunogenicity.

## Conflict of interest

None disclosed.
